# The Use of Prophylactic Antibiotics in Surgery for Dupuytren’s Disease: A Survey of Hand Surgeons

**DOI:** 10.29252/wjps.9.2.135

**Published:** 2020-05

**Authors:** Murtaza Kadhum, Pierre Sinclair, Claire Middleton

**Affiliations:** 1 Oxford University Clinical Academic Graduate School, University of Oxford, Oxford, England;; 2 Trauma and Orthopedics, Royal Berkshire Hospital, Reading, Oxford, England

**Keywords:** Dupuytren’s disease, Surgery, Prophylactic, Antibiotic

## Abstract

**BACKGROUND:**

National Institute for Health and Care Excellence (NICE) CG74 has set out evidence-based guidance on which types of surgery require antibiotic prophylaxis. Our aim was to establish what the current practice for antibiotic prophylaxis in Dupuytren’s surgery is amongst hand surgeons in the United Kingdom, through the British Society for Surgery of the Hand (BSSH).

**METHODS:**

Permission was granted for our online survey to be distributed to BSSH hand surgeons via consecutive BSSH e-bulletins. Hand surgeons who did not perform fasciectomy or dermofasciectomy were excluded from the study.

**RESULTS:**

There were 64 respondents, represented an estimated 7.4-7.8% of membership. Eleven percent of respondents used antibiotics for fasciectomy, with an increasing trend towards revision surgery and dermofasciectomy. Over 30% prescribed them for revision dermofasciectomy. Dupuytren’s surgery was classified as clean, non-prosthetic and uncomplicated which NICE CG74 suggestions did not require antibiotic prophylaxis.

**CONCLUSION:**

This study highlighted variation in practice amongst hand surgeons in the United Kingdom. Further consultation to create guidelines for hand surgery may help guide members and reduce potentially unnecessary prophylactic antibiotic use.

## INTRODUCTION

The advent of penicillin discovery by Alexander Fleming in 1928 revolutionized the management of patients with infections caused by organisms such as *Staphylococcus aureus*. Subsequent studies in abdominal surgery proved its efficacy to reduce the post-operative infection rates when used prophylactically.^1^^,^^2^ From the late 1940s; however, drug resistance to penicillin was encountered as mutations resulted in these organisms acquiring the genes to produce β-lactamase;^3^ thereby, rendering Penicillin ineffective. This led eventually to the development of newer antibiotics as well as tighter controls on their use.

Dupuytren’s disease is an autosomal dominant condition causing progressive fibromatosis of the palmar fascia of the hand. If the disease progresses, the contracture that ensues may cause progressive flexion of the affected digit. Current evidence supports surgical treatment of this condition via percutaneous needle fasciotomy, collagenase injection directly into the cord (in a two-stage process), fasciectomy or dermofasciectomy. To reduce the risk of surgical site infections (SSI), the National Institute for Health and Care Excellence (NICE) recommends the use of antibiotic prophylaxis in clean surgery that involves placement of a prosthesis or implant, any clean-contaminated surgery or any contaminated surgery.^4^

Currently, there is no specific guidance surrounding the use of prophylactic antibiotics in fasciectomy or dermofasciectomy. In a recent meta-analysis of 2578 patients, the use of pre-operative intravenous prophylactic antibiotics versus placebo or no antibiotics was compared for simple hand injuries. They found no difference in the post-operative infection rates between the two groups.^5^ A single-center retrospective review of 8,850 elective hand cases which also compared the use of pre-operative intravenous prophylactic antibiotics with no antibiotic prophylaxis did not find a significant difference in the incidence of SSI between the groups.^4^

Despite this, there has been anecdotal evidence that pre-operative intravenous prophylactic antibiotics are currently being used prior to surgery for Dupuytren’s disease. It is important to note that neither of the aforementioned studies, nor any other currently in the literature, has compared the use of pre-operative prophylactic antibiotics in fasciectomy or dermofasciectomy.^4^ The aim of this survey was to establish what the current practice is amongst Hand Surgeons in the United Kingdom, through the British Society of Surgery of the Hand (BSSH). 

## MATERIALS AND METHODS

Permission was granted for our online survey consisting of 9 questions (Table 1), to be distributed to BSSH members. The survey was distributed via their bi-monthly e-bulletin and remained live for 4 months (September to December 2018). Its link was included in consecutive BSSH e-bulletins in both October and December. It is estimated that the e-bulletin is distributed via email to 800 BSSH members and associate members who performed surgeries of the Hand. This survey was designed to ascertain whether BSSH members routinely used antibiotic prophylaxis prior to their primary or secondary fasciectomy or dermofasciectomy. 

**Table 1:** Summary of responses to the PAID survey

**Questions**			
Q1.	Please indicate which description most applies to you	Hand surgeon - Plastic surgery	17
		Hand surgeon - Trauma orthopedics	43
		Hand surgeon - Other*	3
Q2.	How many years have you been in practice as a Hand surgeon?	1-5	18
		6-10	13
		11-15	10
		16-20	4
		21-25	12
		>25	6
Q3.	Do you currently perform surgery for Dupuytren’s disease?	Yes	62
		No	1
Q4.	Approximately how many surgeries in one year do you perform for Dupuytren’s disease?	Less than 10	3
		10-20	11
		21-30	14
		more than 30	34
Q5.	Do you use antibiotic prophylaxis for…	Yes	No
	Fasciectomy	7	55
	Dermofasciectomy	15	47
	Revision fasciectomy	11	51
	Revision dermofasciectomy	19	43
Q6.	When did you last treat a patient for infection following a fasciectomy or dermofasciectomy?	Never	14
		Within the last month	3
		> 1 month ago, but within the last 6 months	8
		> 6 months ago, but within the last year	11
		More than 1 year ago	26
Q7.	Please give details of any treatment required…	Outpatient antibiotics**	35
		Outpatient treatment not specified	1
		Inpatient intravenous antibiotics only	1
		Surgery***	4
		Unable to recall or declined to comment	7
Q8.	If you know your infection rate for this surgery, please comment below****	Less than 1%	14
		1-5%	10
		6-10%	0
Q9.	Have you had a change in practice regarding antibiotic use during surgery for Dupuytren’s disease?	yes	no
	Hand surgeons with previous case(s) of post-operative infection	4	44
	Hand surgeons who have not had a previous case of post-operative infection	0	14

^*1^BSSH member was an Accident and Emergency Consultant with a special interest in Hand surgery. ^*1^BSSH member was exclusively a Hand Surgeon. **^7^Additionally had regular change of dressings and wound care on an outpatient basis. ***^3^Returned to theatre for a wound washout. Two of these were subsequently treated with inpatient IV antibiotics and the other with oral antibiotics. ***^1^Returned to theatre for drainage of hematoma and subsequently completed a course of oral antibiotics. ****Other responses (n=26) reflected uncertainty.

Therefore, hand surgeons (n=1) who did not perform either of these surgeries were filtered and excluded from the study. A written consent was provided from each patient. The study was approved in the institution ethics committee. To reduce the risk of obtaining incomplete data, it was made compulsory to provide an answer for each question in order for respondents to complete the survey. Secondary outcomes focused on recollection of any previous infective complication following any of these surgeries, how this was managed, and whether or not a change in antibiotic prescribing practice occurred as a consequence. 

In addition, demographic information about the respondents was collected and was summarized in Table 1. Correlation analysis (Point Biserial Correlation) using SPSS software (IBM 2017) was performed between years of clinical practice as a Hand surgeon and the use of antibiotics. Neither the respondents nor BSSH requested or were granted access to data collected.

## RESULTS

Sixty-three BSSH members responded to the survey, which represented an estimated 7.4 to 7.8% of their membership. All except for 3 declared their primary specialty as either trauma and orthopedics (n=43) or plastic surgery (n=17). Over 75% performed more than 20 surgeries per year to treat Dupuytren’s disease. Hand surgeons were more likely to use prophylactic antibiotics in revision surgery and for dermofasciectomy, with over 30% administering them prior to revision dermofasciectomy. 

Only one surgeon indicated that they used prophylactic antibiotics for all fasciectomy surgeries (primary and revision); however, not for dermofasciectomy. Whilst, there was an even split amongst surveyed plastic surgeons who used prophylactic antibiotics (8 vs. 9 plastic surgeons), Trauma and Orthopedics hand surgeons were more likely to perform these surgeries in the absence of antibiotic prophylaxis (11 vs. 33 orthopaedic surgeons). Of all polled, who had less than 7-year experiences as a hand surgeon (33% of respondents), 86% and 82% used antibiotic prophylaxis for fasciectomy and revision fasciectomy, respectively. 

Utilizing the Point Biserial Correlation in SPSS software (Version 21, Chicago, IL, USA), a significant negative correlation was found between years of practice and use of antibiotics in fasciectomy and revision fasciectomy (r_pb_=-0.28 and r_pb_=-0.38 respectively, *p*<0.05). No significant correlation was identified for dermofasciectomy and revision dermofasciectomy, or number of surgeries per annum for Dupuytren’s disease and prophylactic antibiotic use. 

In response to the question aiming to ascertain the last time any of the polled hand surgeons treated a Surgical Site Infection (SSI) in their patient following surgery for Dupuytren’s disease, 4 of 20 (20%) who routinely used antibiotics and 10 of 42 (24%) who did not routinely use antibiotics reported that they have never had this post-operative complication. Of the 48 respondents that had a patient with a SSI, 73-75% commented that the treatment of the SSI was on an outpatient basis and consisted of a course of oral antibiotics with regular change of dressings. Four hand surgeons mentioned that their most recent cases of infection had to be managed with a return to theatre; 3 had a washout and the other had an evacuation of hematoma. All 4 subsequently received a course of either intravenous of oral antibiotics.

## DISCUSSION

NICE Quality Standard 49 published in October 2013 advised against the use of routine antibiotic prophylaxis during clean uncomplicated surgery, unless it involves the insertion of an implant or prosthesis.^4^ This is largely due to evidence obtained from several randomised control trials (RCT) done across different specialties showing no statistically significant differences in post-operative SSI rates, following the use of antibiotic prophylaxis in such circumstances. 

However, its use can lead to detrimental effects, such as causing *Clostridium difficile*-associated diseases, development of multi-drug resistance organisms and hypersensitivity.^4^^,^^6^ In light of the need to be prudent when using antibiotics, our survey aimed to learn about current trends in prophylactic antibiotic prescribing in surgery for Dupuytren’s disease. Through this study, we learned that up to 32% of hand surgeons use peri-operative prophylactic antibiotics during primary and revision fasciectomy and dermofasciectomy. Interestingly, our results suggested that experienced surgeons tended to utilize prophylactic antibiotics for dermofasciectomy and revision dermofasciectomy, whereas less experienced surgeons used antibiotics in all surgeries for Dupuytren’s disease.^7^

This is despite the recommendations set out by NICE CG74, which is based on evidence refuting the efficacy in reducing post-operative SSI rates when compared to placebo, and when used in clean non-prosthetic uncomplicated surgery. There are reports of similar findings from studies of prescribing trends in clean hand surgeries in the United States. A national survey was conducted to identify prescribing trends of prophylactic antibiotics in patients who either had a carpal tunnel release, trigger finger release, de Quervain release or wrist ganglion excision surgery from 2009 to 2015. They found that of 305,946 of patients who underwent these surgeries, an average of 13.6% of patients were given antibiotic prophylaxis.^7^

More interestingly, their trend analysis highlighted a statistically significant progressive increase in the prescribing of peri-operative prophylactic antibiotics during that period in the aforementioned surgeries, except for trigger finger release (10.6% to 18.3%, *p*<0.001).^5^ In light of these trends, there may be a need for large epidemiological studies to re-evaluate the incidence of SSI following clean non-prosthetic uncomplicated surgery. If infection rates are proven to be low as currently reported, then the overall effectiveness of current guidance and its implementation should be reviewed. 

A single-center retrospective review from the Department of Plastics and Reconstructive Surgery in Pittsburgh USA compared the rates of SSI following clean hand surgery in patients who received peri-operative antibiotics prophylaxis versus patients who did not. It was shown that 8,850 patients’ records were included in their analysis from October 2000 to October 2008, of which 31% had received peri-operative prophylactic antibiotics.^6^ Their results indicated that the use of prophylactic antibiotics did not significantly alter the rate of SSI, postoperatively (0.54%) when compared to those not given prophylaxis (0.26%).^6^

More recently, a meta-analysis was conducted to establish whether there was sufficient evidence to support the use of peri-operative prophylactic antibiotics to manage simple hand injuries. The majority of studies included in their analysis classed simple hand injuries as ‘class III’ or ‘contaminated’. ‘Class IV’ or ‘dirty wounds’ were excluded, leaving 2,578 patients from 13 studies. In similar fashion, they found no significant reduction in SSI rate in patients who received antibiotic prophylaxis compared to placebo or no prophylaxis (RR 0.89, 95% CI 0.65 to 1.23, *p*=0.49%).^5^

This finding was also maintained when restricting their meta-analysis to the higher quality five double-blind RCTs involving 864 patients (RR 0.66, 95% CI 0.36 to 1.21, *p*=0.18).^5^ Perhaps, there are a subset of patients who would benefit from receiving peri-operative prophylactic antibiotics prior to their surgery, regardless of classification of degree of wound contamination. A couple of respondents commented that in hindsight, they should have given prophylactic antibiotics to their patients who went on to develop a SSI. 

The mentioned risk factors included patients who smoked and those who were ill-kempt. Bykowski *et al* performed a subgroup analysis to identify whether gender, smoking status, diabetes mellitus or duration of procedure had an impact on the risk of developing a post-operative SSI. They concluded that antibiotics were not protective, though recognized the limitations of their analysis due to the small number of patients in each subgroup and low overall SSI rates.^6^

The low response rate represents a limitation to our survey. Initial requests for our survey to be distributed via direct emailing of the link to each BSSH member was declined due to the new legislation surrounding ‘General Data Protection Regulations’ which came into effect in May 2018. Despite the deliberately low number of questions to reduce the risk of ‘survey fatigue’ and appearing in the BSSH e-bulletin for 2 consecutive issues, we only received 63 responses. The authors believe that this low response rate which represented an estimated 7.4% of BSSH membership is a direct consequence of the change in legislation, as response to the survey would significantly depend upon the proportion who routinely read their e-bulletin.

In our survey, one respondent indicated that prophylactic antibiotics was used for all fasciectomy surgeries (primary and revision); however, not for dermofasciectomy. Though it is not clear, given the prescribing trends and design of this survey, the authors believe this anomaly may be due to the survey not specifically establishing whether the respondents performed fasciectomy separately from dermofasciectomy, and perhaps in this case, the hand surgeon may not have routinely performed dermofasciectomy.

Despite the growing evidence that peri-operative prophylactic antibiotics are not significantly efficacious in reducing the incidence of SSI in ‘class I’ clean non-prosthetic uncomplicated surgery, our survey of BSSH hand surgeons highlighted the continued trend of prescribing these for patients who underwent surgery to manage their Dupuytren’s disease. These trends were also observed in the United States. Although NICE guidance is helpful in general, it may help if Hand Societies led in producing specific guideline for antibiotic use in hand surgery. Further research via a case-control methodology is needed to identify, whether antibiotics may prove useful in reducing SSI in certain patient subgroups, such as smokers or diabetics.

## References

[1] Westerman EL (1984). Antibiotic prophylaxis in surgery: historical background, rationale, and relationship to prospective payment. Am J Infect Control.

[2] Saaiq M, Ahmad S, Zaib MS (2020). Burn wound infections and antibiotic susceptibility patterns at pakistan institute of medical sciences, islamabad, pakistan. World J Plast Surg.

[3] Moellering RC Jr (2012). MRSA: the first half century. J Antimicrob Chemother.

[4] National Institute for Health and Care Excellence (c2017). Surgical site infections: prevention and treatment Clinical guideline [CG74].

[5] Murphy GR, Gardiner MD, Glass GE, Kreis IA, Jain A, Hettiaratchy S (2016). Meta-analysis of antibiotics for simple hand injuries requiring surgery. Br J Surg.

[6] Bykowski MR, Sivak WN, Cray J, Buterbaugh G, Imbriglia JE, Lee WP (2011). Assessing the impact of antibiotic prophylaxis in outpatient elective hand surgery: a single-center, retrospective review of 8,850 cases. J Hand Surg Am.

[7] Johnson SP, Zhong L, Chung KC, Waljee JF (2018). Perioperative Antibiotics for Clean Hand Surgery: A National Study. J Hand Surg Am.

